# A Novel 96well-formatted Micro-gap Plate Enabling Drug Response Profiling on Primary Tumour Samples

**DOI:** 10.1038/srep09656

**Published:** 2015-04-13

**Authors:** Wei-Yuan Ma, Lo-Chang Hsiung, Chen-Ho Wang, Chi-Ling Chiang, Ching-Hung Lin, Chiun-Sheng Huang, Andrew M. Wo

**Affiliations:** 1 Institute of Applied Mechanics, National Taiwan University, Taipei, Taiwan; 2 Institute of Zoology, National Taiwan University, Taipei, Taiwan; 3 Department of Oncology, National Taiwan University Hospital, Taipei, Taiwan; 4 Department of Surgery, National Taiwan University Hospital, Taipei, Taiwan

## Abstract

Drug-based treatments are the most widely used interventions for cancer management. Personalized drug response profiling remains inherently challenging with low cell count harvested from tumour sample. We present a 96well-formatted microfluidic plate with built-in micro-gap that preserves up to 99.2% of cells during multiple assay/wash operation and only 9,000 cells needed for a single reagent test (i.e. 1,000 cells per test spot x 3 selected concentration x triplication), enabling drug screening and compatibility with conventional automated workstations. Results with MCF7 and MDA-MB-231 cell lines showed that no statistical significance was found in dose-response between the device and conventional 96-well plate control. Primary tumour samples from breast cancer patients tested in the device also showed good IC_50_ prediction. With drug screening of primary cancer cells must consider a wide range of scenarios, e.g. suspended/attached cell types and rare/abundant cell availability, the device enables high throughput screening even for suspended cells with low cell count since the signature microfluidic cell-trapping feature ensures cell preservation in a multiple solution exchange protocol.

Drug-based treatments are the most widely used interventions for cancer patients. The treatment provides preoperative tumour size shrinkage and postoperative risk of recurrence elimination. The strategy to decide the most effective regimens, this includes types of anticancer drug, the doses and the combinations, for patients is still based on general empirical information instead of individual anticancer drug response^1^. Having to base on general empirical data could lead to repeats of ineffective drug treatment which can seriously harm patients physically and psychologically, since tumours have shown differently in its genotype and phenotype^2^,^3^,^4^. The difference on these indicators could cause dramatic variation of individual anticancer response. In order to conduct more effective and personal treatment, many approaches have been developed for individual tumour response. The feasibility of individualized drug susceptibility has been shown with *in vitro* testing using different drug sensitivity and resistance assay methods^5^.

Human tumour cloning system (HTCA) predicted the most effective single treatment based on the outcomes of *in vitro* testing on tumour colony-forming units (TFUs)^6^,^7^. Moreover, in order to have higher cloning efficiency, a modified HTCA, capillary cloning system (CCS) was also developed^8^,^9^. In order to conduct multi-concentrations of different drugs assays on tumour specimens, the cell-death assay was developed to provide information of cell apoptosis under combinations of drug treatment. For example, differential staining cytotoxicity (DiSC) assay, allowed multi-concentrations of different drugs testing on fresh tumour specimens^10^,^11^,^12^. This assay is capable of discriminating drug effects of cancer cells versus normal cells. Meanwhile, researchers not only focused on developing assays to find most effective treatment but also developing assays to exclude treatment that least effective for patients. The extreme drug resistance assay (EDR) was developed to meet this need^13^,^14^. On the other hand, in order to build assays which are processed in environment which is more similar to original living condition in host, *in vivo* assays were also developed for the prediction of drug sensitivity. Subrenal capsule assay (SRCA) is one of these assays which conducts drug response test by treating drugs to mouse' kidneys with tumour pieces inserted^15^,^16^. In addition, histoculture drug response assay (HDRA) maintained the cell-cell contact as in their native 3 dimensional tissue environment enabling accurate drug sensitivity assay^17^,^18^. Many assays were developed and have shown excellent correlation between in vitro resistance and clinical resistance of the patient's tumour to same regimen.

In addition to those conventional assays stated above, microfluidic technology had also shown feasibility of anticancer drug screening on cell line. Microfluidic drug perfusion system was developed for continuous drug treating environment^19^,^20^,^21^. Forming micro 3D spheroids to further understand drug resistance of cancer cells in tissues was also presented^22^,^23^,^24^. High content drug screening allowed parallel multi-concentration or multi-type of anticancer drug treatment^25^,^26^. Although these microfluidic devices have been proven as an alternative for future anticancer drug assay, some problems still remained unsolved when regarding with the primary tumour samples.

The common encountered problem is the size of specimen not always sufficient for a complete test. In order to obtain the sufficient number of testing cells, some conventional assays spent days culturing primary cells and a complete test took 1 to 2 weeks. Due to these major obstacles, a more rapid and less cell demanding method with high accuracy is still desirable.

Here, we describe the operation and physical principles of micro-gap plate (MGP) and applied it for individual tumour response profiling while demanding only 9,000 cells in single reagent test. This device enables rapid in vitro individual drug response profiling. Now that the cells demand in this method is extremely low comparing with other methods, there would be no need for long-term primary culturing before drug test. As a result, the duration of a complete testing lasted only 3 days. In addition, a SOP was developed for uniform quantity of cell seeding in each well since cell-cell interaction and reagent-to-cell ratio may considerably affect the outcome of drug response. Moreover, common encountered problems with microculture were also discussed in this paper for stable drug treatment condition, such as, prevention of evaporation, stable concentration of solution and cells loss minimization. Then, anticancer drug response on breast cancer cell lines were performed on MGP to prove the feasibility of the device. Finally, drug response profiling of tumour samples by MGP were presented and discussed.

## Results and Discussion

### Design of the micro-gap plate

**Figure 1:**
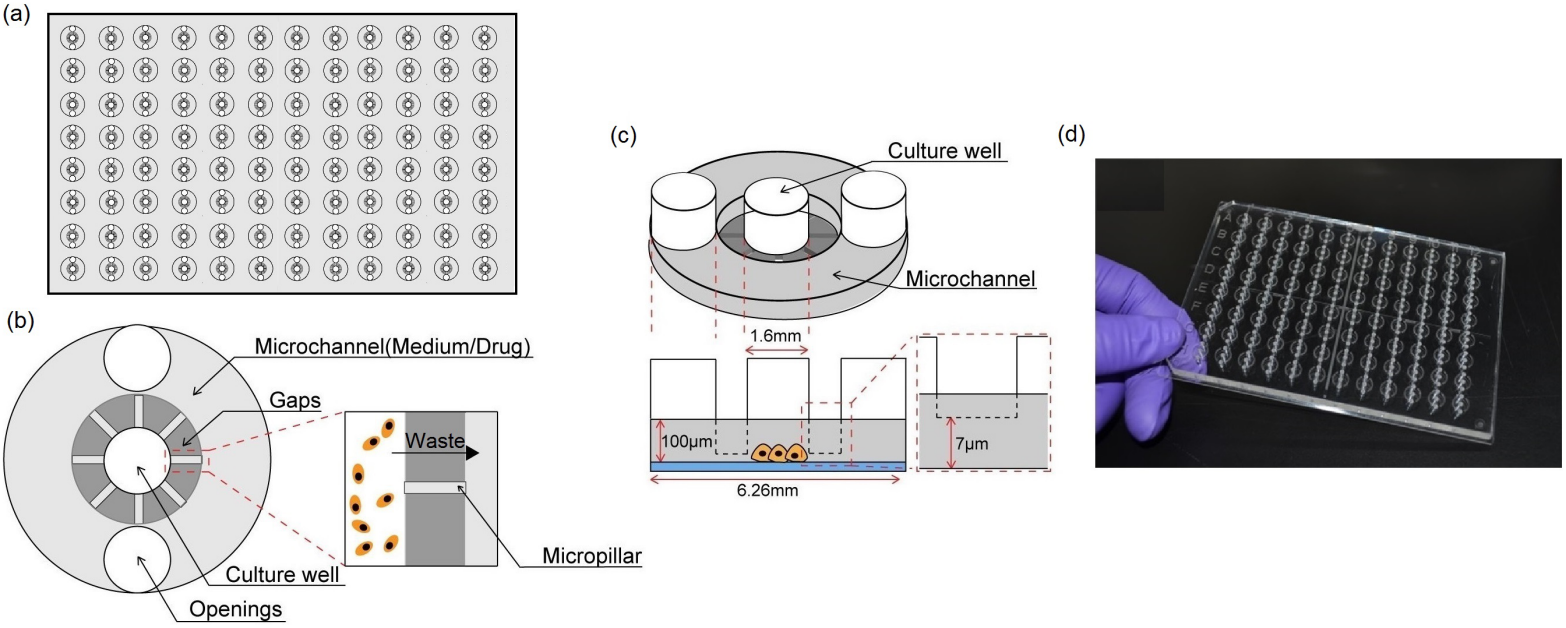
The micro-gap plate (MGP). (a) Illustration of top view of the MGP. 96 units were included in one MGP, the units are arranged in the standard 96 well plate format (8 rows, 12 columns, and 9 mm apart in both directions). (b)Top view of a single unit. A unit consists of three regions; culture well, microchannel and micro-gaps. Culture well was the region for cell seeding and culturing prior to drug treatment. Microchannel was designed to contain different reagents while operating tumour response assay. Gaps were connection between the two regions for the diffusion of nutrients/drug into culture well and waste out to microchannel. (c) 3D structure of MGP unit. The height of gaps was 7 μm, which was slightly smaller than the diameter of single cells to prevent cell loss during solution exchange. The height of the microchannel was 100 μm. (d) Photo of the micro-gap plate.

In this work, we designed a micro-gap plate (MGP), shown in Fig. 1, to conduct individual anticancer drug response assay with few cells demanded and less external instruments requirement. Our design concept, associated with the compatibility with existing scanning and liquid handling instruments, a user-friendly interface, and reducing cell loss, is presented as following.

Compatibility with existing scanning instruments allows more rapid fluorescent data detection while maintaining high-quality image signaling. Conventional polycarbonate was applied as the material of MGP, which had been proven with high optical transparency, ensured the quality of detecting images. This would improve the accuracy of cell viability assessment after drug treatment. Moreover, applying conventional material was able to further lower the cost of fabrication than that of other material-based (e.g. PDMS) microfluidic devices.

A user-friendly interface provides a much easier way to operate microfluidic device. In previous years, many microfluidic devices had shown outstanding efficiency on their functionality. However, many of them required lots of external instruments and internal on-chip components to operate which reduced the usability. In order to eliminate the requirement of external instruments, we designed a tubing-free microfluidic device which can be operated with conventional lab-used pipettes. In this case, solution can be directly introduced into and removed from device by pipettes instead of by other complicated and expensive instruments (i.e. syringe pumps and pressure supplies). This would reduce the complexity of the operation which also provided a user-friendly interface not only for engineers but also researchers at fields of medical and biological researches. Furthermore, the specified dimension of MGP unit enables the compatibility with commercial automated liquid handling workstations for rapid solution introduction and drug treatment.

Cell loss should be reduced since the quantity of primary cells from each specimen is extremely low comparing to that from cell line. In this work, the geometry of MGP units was optimized to conduct no/low cell loss while processing every procedure. The MGP unit can be divided into 3 regions: 1) culture well, 2) micro-gaps and 3) microchannel (Figure 1(b)). 3 holes on a unit were design for different purposes. Two holes on microchannel were openings for solution removing and introducing while the middle one served as culture well (Figure 1(b)). The two holes on microchannels would reduce bubbles occur in units since air could be flushed out from the other end while introducing solution through one of them. In addition, the surrounding microchannel with 2 openings was also applied as reservoir for containing culture medium, anticancer drug and fluorescent dye while conducting cell seeding, drug treatment and viability assessment respectively. The culture well was the region where cells were plated and cultured. Cell suspension was directly introduced into this region and the density of cells in each well was optimized (1,000 cells/well) for sufficient cell-cell interaction^27^. On the other hand, in order not to directly contact with cells while solution exchange, solution was removed through surrounding microchannel and introduced to culture well respectively. For easily removing solution from microchannel, culture well and microchannel was connected by 7 μm high gaps (Figure 1(c)). The height of gaps was slightly smaller than that of a single cell so cells can be well conserved in culture well during solution exchange for accurate assessment on cells. Micropillars were fabricated to prevent micro-gap from collapsing during thermal-bonding fabrication of MGP. Details of MGP fabrication and sterilization were presented in Supplementary Information (see Supplementary Figure S1 & S2).

### Procedures for drug response profiling on tumour sample using MGP

The overall procedure could be divided into three sequential steps: cell seeding, drug treatment and viability assessment. All preparations of solution and exchange were done by conventional lab-used pipette. **Step.1 Cell seeding** Cell was two-step diluted into desired density (16.7 cells/0.1 μl) in culture medium (SOP for two-step dilution please see Supplementary Information). The cell suspension was then sequentially introduced into culture wells (1,000 cells/well). After cells were successfully introduced into collagen pre-coated culture wells, 10 μL of culture medium was then introduced into each microchannel to create a normal culture environment. Between microchannel and culture wells were micro-gaps for diffusion of nutrients in culture medium. The height of gaps were extremely low comparing to that of microchannel so that shear stress can be reduced while introducing culture medium and other solution in following steps since huge shear stress could cause cells detachment^28^. Cells seeded MGP was covered with a standard 96well cover and sealed with parafilm to minimize evaporation. The closed microculture system was then incubated at 37°C, 5% CO_2_ overnight prior to drug treatment. **Step.2 Drug treatment** Cisplatin (Bristol-Myers Squibb) and docetaxel (Taxotere) were used in this work. The platinum agents are commonly used for the treatment of ER positive breast cancer. Recently, there is renewed interest in further investigating the efficacy in triple negative breast cancer. On the other hand, taxane agents are used for platinum-resistant cancers. The gradient of drugs used was prepared externally in Eppendorf tubes using a three-step approach. First, a full range of drug dosage was made from undiluted drug to much-diluted dosage (e.g. 1X, 2X, 4X, ¼, 128X dilution). Second, from the full range of dosage obtained in the previous step, pilot tests on cellular samples were commenced to determine the likely range of dosage within the full dosage range. Last, using the refined dosage range in Step 2, comprehensive testing on cells was studied. In the drug preparation step, each drug was mixed with culture medium then diluted into 3 or 4 concentrations. The culture medium in MGP was then manually replaced by the drug-mixed medium via a pipette for 24 hours of treatment. In order to prevent bubbles occur during solution replacement, culture medium in MGP unit was replaced by cycles of partial replacement^29^. The repeats of partial solution replacement was optimized as stated in Supplementary Information (see Supplementary Figure S3). During drug treatment, pictures of treated cells were taken by microscope (Olympus, IX71) every 12 hours. **Step.3 Cell viability assessment** After 24 hours of drug treatment, viabilities of each well were then determined by tri-fluorescent staining. A fluorescent mixture of PE (conjugated with EpCAM), 4 μM Hoechst (33342, Invitrogen) and 0.1 μM SYTOX (SYTOX®, Invitrogen) was fresh prepared for identification of cell viability. Different concentrations of drug were replaced by fluorescent mixture to stain epithelial cells and dead cells. Bright field images, red fluorescent images (emission of PE, epithelium cells), blue fluorescent (emission of Hoechst, all cells) and green fluorescent (emission of SYTOX, dead cells) of cells images were captured. Quantity of cells was automatically counted by journal written in MetaMorph to estimate viability from composed images^30^.

### Cell conservation after solution exchange

**Figure 2:**
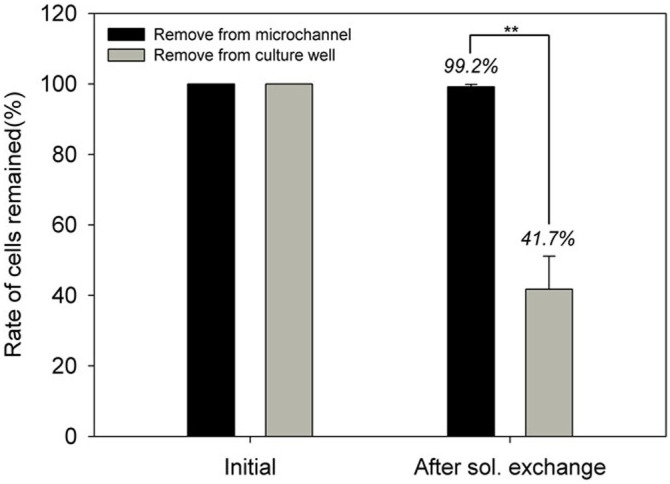
The cell conservation rate on the MGP after solution exchange. After sufficient repeats of solution exchange, rate of cells remained in culture wells was compared in this figure. Once solution was removed from culture wells directly, rate of cells remained was dropped to 41.7%. By removing solution from surrounding microchannels, rate of remained cells was up to 99.2%. (Data were mean ± standard deviation (SD), n = 3, Student's t-test, P value = 0.006).

Figure 2 showed the cell conservation rate during solution exchange and a comparison with directly removing solution from culture well is also presented in the same figure. The solution exchange was processed few repeats in order to sufficiently replace the solution. Figure 2 showed that after 3 repeats of solution exchange, cells were conserved in culture wells of MGP units. Up to 99.2% of cells were conserved in culture wells which showed statistical insignificance with the original quantity. On contrast, cells in wells which solution was directly removed from culture well could be conserved 41.7%, which is statistical significance with original quantity (p value = 0.006). By replacing solution from microchannels on MGP units, cells can be conserved which would also benefit cell-cell interaction. The cell quantification in MGP units were obtained by a microscopy automation & image analysis software, MetaMorph. A correlation between manual quantification and automatic one by MeraMorph was also done to improve the confidence on machinery quantification (see Supplementary Figure S4).

### Stability of solution concentration during long-term incubation

**Figure 3:**
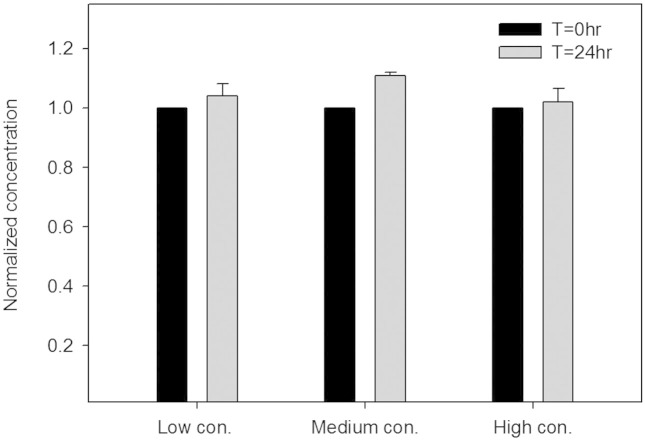
The concentration of solution before and after 24 hour incubation. Low, medium, high concentration was 12.5, 25, 50 μM, respectively. No statistical significance was found between t = 0 hour and t = 24 hour, suggesting that the concentration of solution was stable during 24 hours incubation. (Data were mean ± standard deviation (SD), n = 3, Student's t-test, P > 0.05).

The concentration of reagent in units could have crosstalk with each other due to unwanted condensation in culture system^31^. To examine if evaporation considerably happen on the MGP, a series concentration of fluorescent dye (12.5, 25, 50 μM, FITC) was applied to test the shift on concentration during long-term incubation. After dye introducing, the plate was covered by a standard 96-well plate cover and sealed by parafilm. In addition, parafilm is permeable to CO_2_ and O_2_, moreover, its low permeability to water vapor will help the MGP system maintain relative humidity. After 24 hours of incubation, we loaded the same batch of FITC to compare if there was any shift on concentration during incubation by analysing the fluorescent intensity of pictures. The conditions of pictures capturing were fixed before and after incubation. The comparison of 0 hour incubation and 24 hour incubation was conducted by Student's t-test and showed in Figure 3. No statistical significance was found between t = 0 hour and t = 24 hour, suggesting that the concentration of solution was stable during 24 hours incubation.

### Drug response profiles of cancer cell lines on MGP and 96-well

**Figure 4:**
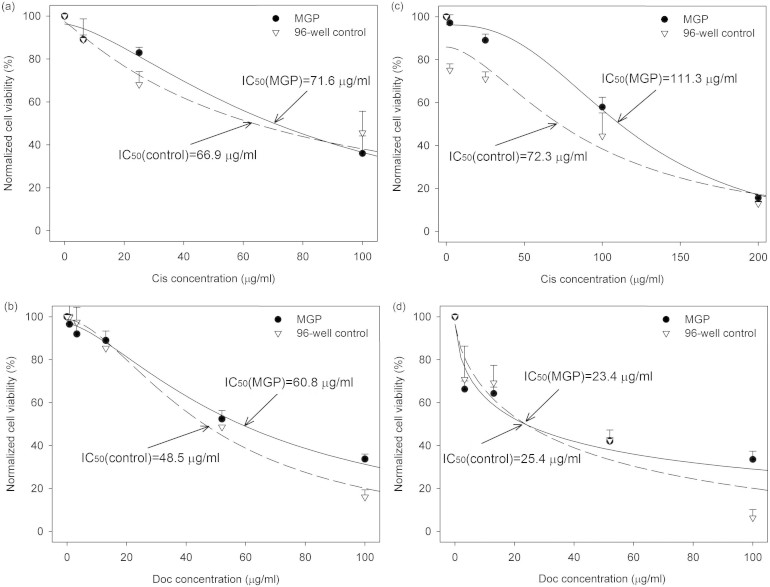
Drug response profiles of (a) cisplatin and (b) docetaxel by MGP and on 96-well plates after 24 hour drug treatment on MCF7 cells; (c) cisplatin and (d) docetaxel on MDA-MB-231 cells. Cells viability in different units of microfluidic device with different concentration drug treatment was normalized against that of untreated cells. No statistical significance in the dose-response between MGP and 96-well plate was found. The dashed line represents the response profile conducted in 96-well plates while solid represents that in MGP. (Data were means ± standard error of the mean (SEM), n = 3, Student's t-test, P > 0.05).

MCF7. Morphology of cells during the drug treatments was showed and discussed in Supplementary Information (see Supplementary Figure S5 and S6). Response profiles of cisplatin and doectaxel by MGP and on 96-well plates obtained after 24 hour drug treatment were presented in Figure 4(a) and Figure 4(b). Cell viability of different units treated with different drug concentrations was normalized against that of untreated cells. By applying Student's *t*-test, no statistical significance in the dose-response between MGP and 96-well plates was found. In addition, according to the regression of dose-response, applying a four-parameter logistic equation, IC_50_ values from the two platforms were obtained. The values of IC_50_ obtained from this research were higher than previous studies may be caused by higher cell number-to-drug volume ratio. This could increase the drug resistance since cells were well connected with each other which would further strengthen the cell adhesion with extracellular matrix.

MDA-MB-231. Response profiles of MDA-MB-231 cells treated with cisplatin and docetaxel in different concentrations after 24 hour were presented in Figure 4(c) and Figure 4(d). In cells treated with cisplatin, a difference in values of IC_50_ was found between the platforms. The value of IC_50_ obtained from MGP was larger than that from 96-well plates. The probable cause was that a batch of MDA-MB-231 seeded in the MGP grew considerably faster than the other batches. As a result, the number of cells to a specific area in that experiment was found considerably larger than others. Since the intercellular connection would affect drug resistance of cells, the viability after drug treatment of that batch showed higher than others. Although the values of IC_50_ show differently between the two platforms in cells treated with cisplatin, by applying Student's *t*-test, no statistical significance was found between the two. On the other hand, the values of IC_50_ of cells treated with docetaxel showed similar to each other (23.4 μg/ml in MGP and 25.4 μg/ml in 96-well control).

### Minimum requirement of cell number for cell-based assays using the MGP

**Figure 5:**
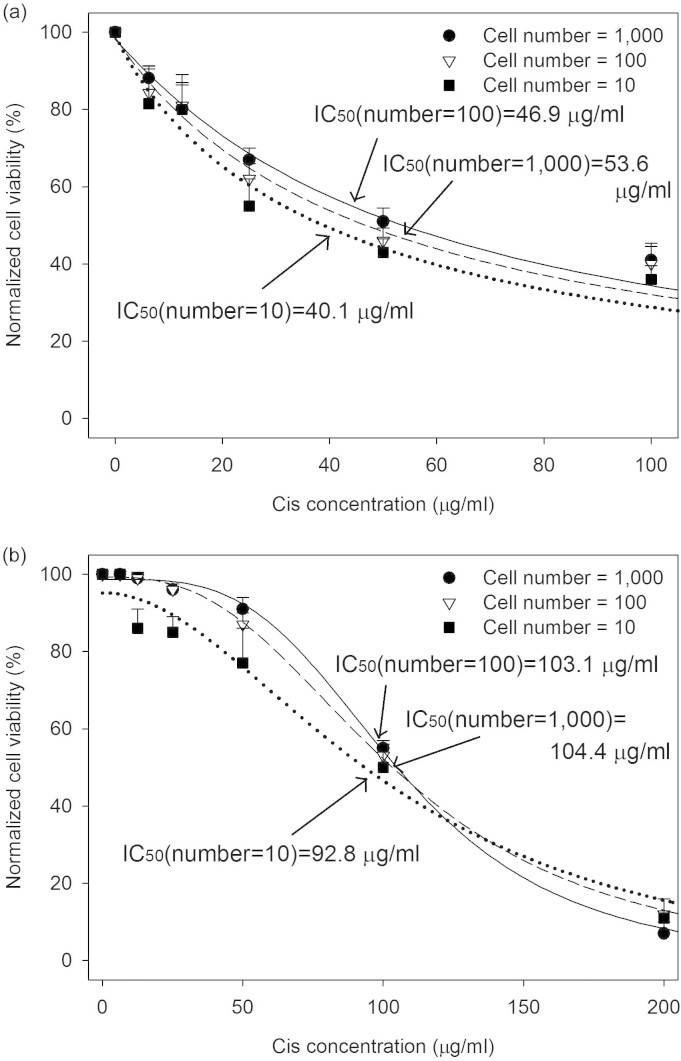
Drug response profiles of cisplatin using different cell number. (a) Response profiles of MCF7 and (b) MDA-MB-231 under three cell number conditions after 24 hour drug treatment on MGP. No statistical significance in the dose-response was found among three cell number conditions in both cell lines. (Data were means ± standard error of the mean (SEM), n = 3, Duncan method, P > 0.05).

To characterize the minimum requirement of cell number of the MGP, three different cell number conditions (i.e. 1,000, 100, 10 cells/well) were used in this experiment. Drug response profiles of MCF7 and MDA-MB-231 under these three conditions were presented in Figure 5(a) and Figure 5(b) respectively. The value of IC_50_ obtained from 100 cells/well condition was similar to that of 1,000 cells/well in both cell lines treated with cisplatin. On the other hand, the value of IC_50_ obtained from 10 cells/well condition was slightly lower than that of 100 and 1,000 cells/well in both cell lines. This shift may result from the lower cell number-to-drug volume ratio and insufficient cell-cell interaction. Nevertheless, no statistical significance was found in three cell number conditions in both cell line, suggesting that the minimum requirement of cell number of the MGP could be down to 10 cells/well which is much lower than conventional well-plates (usually require 200 ~ 800 cells/well in 1536-well). However, it is well known that tumour possess substantial intra-tumour heterogeneity. Therefore, using low cell number to do drug test may not be able to reflect the response from whole tumour. Our experience also confirmed that the cell number should not be lower than 400 ~ 500 cells/well when dealing with patient sample, so we chose to use 1,000 cells/well in following patient sample experiments.

### Clinical pathological features of breast cancer tumour samples evaluated

The pathological features of primary samples collected for MGP assay were built and presented in Table 1. The viability of primary cells after preparation was stained with STYOX and obtained by flow cytometry. The viability was different from patient to patient. In addition, the composition of each sample was also conducted by flow cytometry. The PE conjugated CD326^+^ (Miltenyl Biotec) a type of EpCAM, signals stood for epithelial cells. Based on the analysis of composition, the percentage of epithelial cells was obtained. Furthermore, to better understand the composition of primary samples, cytokeratin (CK) 7/8 (BD Biosciences CA, USA) and CK 8/18/19 (Miltenyi Biotec, Germany) were also applied to the analysis of primary samples since some subtypes of breast cancer cells (Ex. Normal-like) showed low affinity with CD326. To further analyze the correlation between response profiles and pathological features, common clinical used indicators the expression of estrogen receptor (ER), progesterone receptor (PR) and HER2/neu were also collected and presented in Table 1. One sample showed ER positive and HER2/neu negative and another showed HER2/neu positive. The third one showed triple negative.

### Morphology of primary cells of patient sample

#### Patient sample No. 1

**Figure 6:**
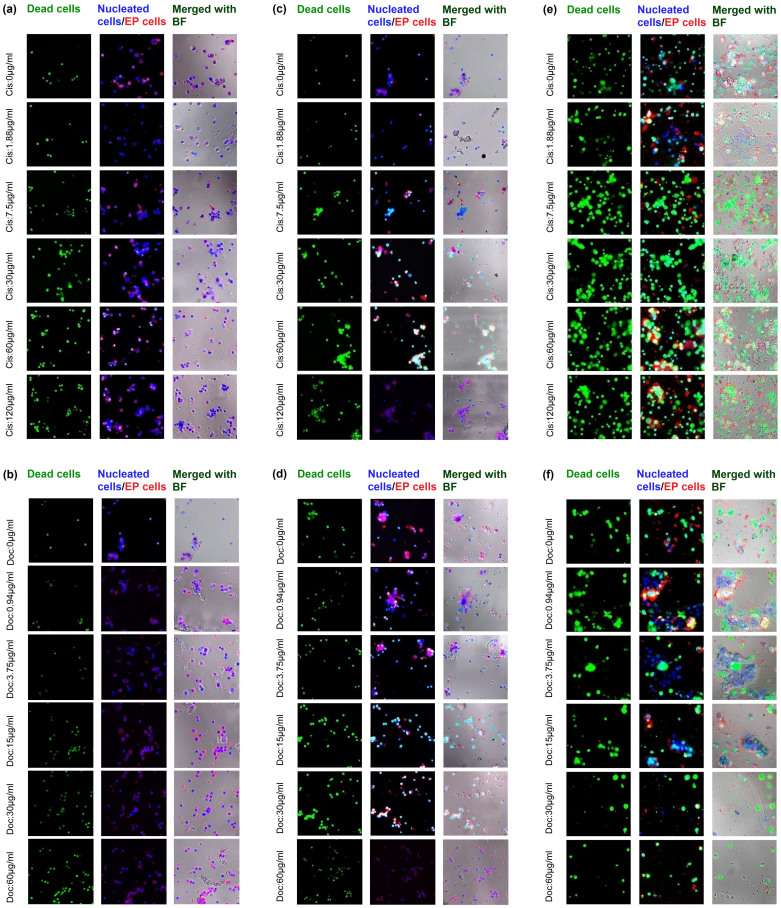
The morphology of cells of patient tumour sample treated with different concentrations of cisplatin and docetaxel. (a) & (b) for sample no. 1, (c) & (d) for sample no. 2, (e) & (f) for sample no. 3, respectively. The morphology of primary cells showed differently comparing with cell lines since many types of cells were in one sample. The blue signals (emission of Hoechst) stand for all cells while green signals (emission of SYTOX) stand for dead cells. The red signals at cell membranes were CD326 conjugated with PE which stands for epithelial cells. Thus, the cells which show purple signals can be viewed as epithelial cells.

The morphology of primary cells no .1 treated with different concentrations of cisplatin and docetaxel were presented in Figure 6(a) and Figure 6(b) respectively. According to the results, the number of green spots (emission of SYTOX) increased while the concentration of anticancer drugs increased which indicated that the cell viability decreased at high concentration anticancer drug treatment.

#### Patient sample No. 2

The morphology of primary cells no. 2 treated with different concentrations of cisplatin and docetaxel were presented in Figure 6(c) and Figure 6(d) respectively. The number of dead cells increased as concentration of cisplatin and docetaxel increased.

#### Patient sample No. 3

The morphology of primary cells no. 3 treated with different concentrations of cisplatin and docetaxel were presented in Figure 6(e) and Figure 6(f) respectively. In this sample, the initial cell viability assessment by flow cytometry showed considerably lower than other two primary samples. Moreover, the loss of plasma membrane integrity was observed in most of cells before drug treatment. Hence, these membrane-compromised cells were prone to undergo apoptosis. This phenomenon can be seen from the number of dead cells at control well (0 μg/ml). In addition, the primary cells of this sample aggregated more than other two in the MGP.

### Drug response profiles of patient samples on MGP

#### Patient sample No. 1

**Figure 7:**
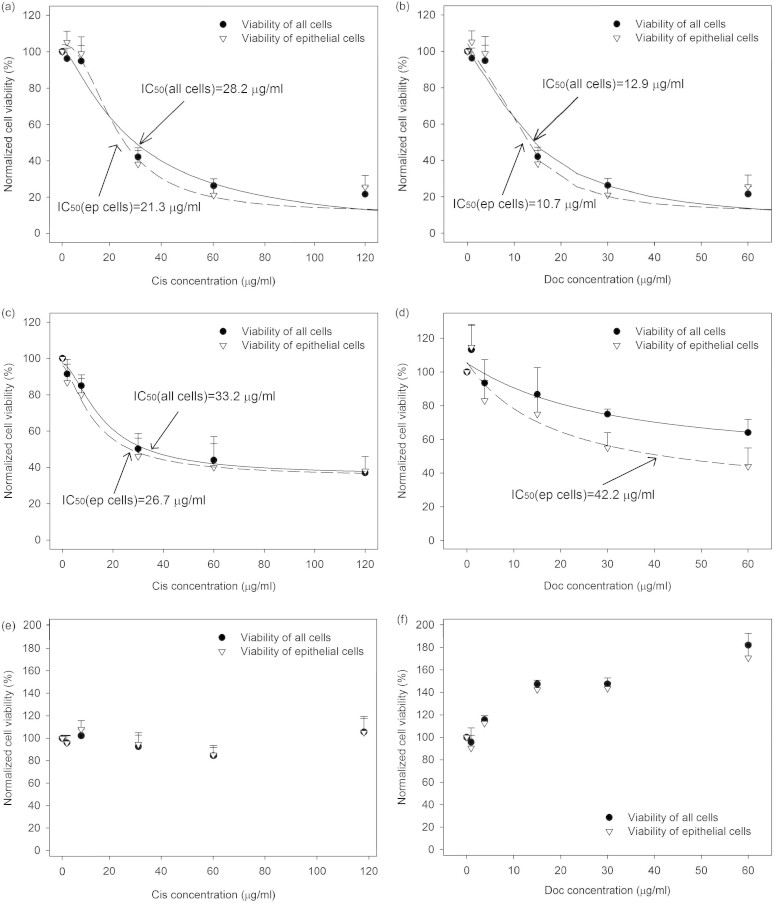
Drug response profiles of cisplatin and docetaxel after 24 hour drug treatment on patient tumour samples. (a) & (b) for sample no. 1, (c) & (d) for sample no. 2, (e) & (f) for sample no. 3, respectively. The cell viability of different concentration of anticancer drug was normalized against to that of untreated cells. The viability of all cells and epithelial cells were presented separately. The dashed line represented the response profile of epithelial cells. (Data were means ± standard error of the mean (SEM), n = 3).

Response profiles of cisplatin and docetaxel on MGP obtained after 24 hour drug treatment were presented in Figure 7(a) and Figure 7(b). Cell viability of cells treated with different concentrations of anticancer drug was normalized against that of untreated cells. According to the regression of dose-response, applying a four-parameter logistic equation, IC_50_ values of primary cells treated with cisplatin and docetaxel were obtained.

#### Patient sample No. 2

Response profiles were presented in Figure 7(c) and Figure 7(d). The values of IC_50_ of the two anticancer drugs were obtained. In addition, primary cells of sample no. 2 showed more resistant to both cisplatin and docetaxel treatment comparing to cells of sample no. 1 treated at the same concentration since the pathological features of sample no. 2 showed Her-2 positive(Her-2 positive cell may show more sensitive to trastuzumab).

#### Patient sample No. 3

Response profiles were presented in Figure 7(e) and Figure 7(f). The values of IC_50_ of the two anticancer drugs were not available. The cell viability at different concentration of cisplatin treatment showed similar to each other. In cells treated with docetaxel, the cell viability increased even at the high concentration of anticancer drug. This may be caused by the initial condition as mentioned above. Thus, the normalized cell viability could even show close to each other. In addition, the cell viability at high concentration of docetaxel could also be caused by this initial condition.

Based on the results of the three primary tumour samples, some experience can be taken into concern to improve the quality of drug response profiling. A threshold of initial cell viability should be built to get available results. The types of patient samples should be selected since different subtypes of breast cancer could show different recurrence and overall survival. The general concentration range for tumour samples should be established. Once the criteria of selecting suitable concentration range of anticancer drugs are established, the quality of results should also be improved. The result obtained from MGP can be further examined by comparing with empirical information.

## Conclusions

We presented a microfluidic device for anticancer drug response profiling using cell lines and also primary tumour samples. The microfluidic device consumed fewer cells comparing with cell-based drug response on 96-well plate. Consequently, drug response using cisplatin and docetaxel on MCF7 and MDA-MB-231 obtained from the MGP showed functionally equivalent with that from 96-well plate control. This suggests that this platform is applicable to drug response assay while the size of tumour sample or the cell supply is limited. In addition, the common encountered problems, evaporation of reagents, crosstalk among wells and insufficient solution exchange, in microculture system were also discussed and further solved by applying suitable approaches. Thus, the device should enable a robust cell-based drug response assay for clinical usage. According to the results of primary tumour samples, the criteria of patient samples can be further established and improved. Conceivably, this microfluidic device may be a powerful tool for clinicians to tailor individualized drug treatment.

## Methods

### Preparation of breast cancer cell lines

Human breast adenocarcinoma cells, MCF7 and MDA-MD-231, were incubated in a culture dish (704001, NEST) at 37°C, 5% CO_2_. The culture medium were Dulbecco's modified eagle medium with nutrient mixture F12 (DMEM/F12) (12400, GIBCO) for MCF7 cell lines and Dulbecco's modified eagle medium (DMEM) (12100, GIBCO) for MDA-MB-231 cell lines respectively. Both of them were added with 10% fetal bovine serum (FBS) (SV304, Hyclone) and 1% penicillin/streptomycin (15140, GIBCO). While processing drug response, cells were firstly detached by 0.05% trypsin with EDTA-4Na (tetra-sodium ethylenediaminetetraacetate) (25300054, GIBCO). The detached cell suspension was then diluted into 1,000 cells/6 μL by the two-step dilution in culture medium for following cell seeding in the microfluidic device. In order to fully simulate the drug response assay on primary tumour, the quantity of cells in each unit of microfluidic device was 1,000 as that of tumour samples. In addition, the procedures of anticancer drug assay in MGP using cell lines were completely the same as on tumour samples. Moreover, in order to eliminate the consistent drug response of cells from same batch, cells for experiments were in different passages but no more than 5 passages in difference.

### Procedures of anticancer drug screening in 96-well plates

Anticancer drug assay was also conducted using conventional 96-well plates as controls. Wells of 96-well plates (NEST) were pre-coated with 100 μg/ml of collagen solution for 1 hour. 17,000 cells in 100 μl were then seeded into wells for a complete control experiment. The quantity of cells in single well was determined to coordinate the same cell-to-substrate ratio as in MGP (1,000 cells/1.766 mm^2^). After cell seeding, cells were incubated in 37°C, 5% CO_2_ overnight prior to drug treatment. The next morning, culture medium in wells was replaced by different concentration of anticancer drug-contained medium. Next, cells were incubated in the medium for 24 hours. Afterwards, the bright-field images were captured as the reference. Then, medium was replaced by 12 mM MTT (M5655, Sigma). The MTT treated cells were placed in 37°C for 3 hours. The MTT assay is based on the cleavage of the yellow tetrazolium salt MTT to purple formazan crystals by dehydrogenases and reductases in live cells^32^. As a result, the formazan crystals were then solubilized by replacing MTT solution with dimethyl sulfoxide (DMSO) (D4540, Sigma) at 37°C for 20 minutes. In the end, a plate reader (MQX 200, BioTek) was used to determine the absorbance of the DMSO at 570 nm, which was used to quantify the number of live cells. The viability in different concentration of anticancer drug was normalized against the untreated cells.

### Preparation of patient's tumour sample

Fresh tumour samples were obtained from surgical specimens of patients in the National Taiwan University Hospital with approval of the NTUH Institutional Review Board. Informed consent were obtained from all patients prior to experiment. All tumour sample experiments were conducted in accordance with government and NTUH Institutional Review Board guidelines. The dissected tumour was suspended in culture medium (DMEM, 5% FBS, 10 ng/mL EGF, 1 μg/mL Cortisol, 5 μg/mL Insulin). Cells were quantified by hemocytometer for following dilution. The total quantities of cells from one specimen were approximately 10^5^ to 10^6^ in 2 mL of culture medium. Cells were then divided into two portions: (1) cells for cytometry to understand the ratio of epithelial cells and initial cell viability, (2) cells for *in vitro* drug response on MGP. A SOP was developed for the preparation of cell suspension. In short, cells suspension was diluted into 16.7/0.1 μL. In addition, to be more accurate to this final number, we used two-step dilution and check the density after each dilution. The expected number of cells to each well was 1,000. Thus, the final volume of cell suspension for each well would be more flexible (6 to 8 μL) since it depended on the final concentration of cell suspension.

### Flow cytometry analysis

Cells from tumour sample were divided into aliquots, single-stained with SYTOX green (Invitrogen, Carlsbad, CA, USA), anti-CD326-PE (Miltenyi Biotec, Germany), anti-Ber-EP4-FITC (Dako, Germany), anti-CK7/8-FITC (BD Biosciences CA, USA) and anti-CK8/18/19-FITC (Miltenyi Biotec, Germany) for 10 min. Stained cells were washed and resuspended in PBS. Non-stained and each single-stained sample were prepared for fluorescent compensation. For all experiments, analysis of cells used a FACSCalibur instrument (BD Bioscience, CA, USA) and FCS Express software (De Novo, Los Angeles, CA, USA).

### Immunohistochemistry of ER, PR and Her2/neu

Immunohistochemistry results of ER, PR and Her2/neu were obtained from the Department of Pathology in National Taiwan University Hospital. Immunohistochemistry staining permitted the detection of ER, PR and Her2/neu within sections from formaldehyde-fixed and paraffin-embedded tissues. The percentage of ER+ and PR+ were individually demonstrated. In Her2/neu analysis, 0 contained no immunostaining, and 1+ indicated weak immunostaining of less than 30% of tumour cells. Results of 2+ was considered as a moderate complete membrane staining which was observed in >10% of tumour cells. Uniform intense membranous staining of >30% of tumour cell nuclei was considered 3+ positive. We determined the results of 0 to 1+ as negative and 3+ as positive. Otherwise, Her2/neu results of 2+ were considered as negative results unless further verified by fluorescent in-situ hybridization (FISH).

## Author Contributions

W.Y.M. and C.H.W. developed the MGP for drug response profiling on tumour samples, performed cell line experiments, and wrote the manuscript. L.C.H. and C.L.C. performed patient tumour sample experiments. C.S.H. and C.H.L. supplied tumour specimens. A.M.W. provided overall guidance and critically reviewed the manuscript.

## Supplementary Material

Supplementary Information Supplementary Information

## Figures and Tables

**Table 1:** Pathological features of breast cancer tumour samples evaluated

Patient No.	Age	Initial Cell viability	Epithelial cells	ER+	PR+	Her2/neu+	Ki-67+
1	47	79.9%	CD326+:76.7%	>90%	3–5%	0+/3+	15–30%
			Ber-ep4+:80.0%				
2	47	54.9%	CD326+:67.2%	>90%	>90%	3+/3+	20–25%
3	73	29.9%	CD326+:48.8%	-	-	1+/3+	N/E
			Ber-ep4+:45.6%				
			CK7/8+:47.4%				
			CK8/18/19+:47.8%				
