# Supplementary material for: A Novel 96well-formatted Micro-gap Plate Enabling Drug Response Profiling on Primary Tumour Samples
Source: Sci Rep. 2015 Apr 13;5:9656. doi: 10.1038/srep09656 (PMC4394194; doi:10.1038/srep09656)
Supplement: Supplementary Information [file srep09656-s1.pdf]

Supplementary Information for

A Novel 96well-formatted Micro-gap Plate Enabling Drug Response Profiling on Primary Tumour Samples

Wei-Yuan Ma, Lo-Chang Hsiung, Chen-Ho Wang, Chi-Ling Chiang, Ching-Hung Lin, Chiun-Sheng Huang & Andrew M. Wo

Fabrication and sterilization of the device

The Micro-gap Plate was composed of two components, as shown in Supplementary Figure S1(a), the top part with 96X3=288 holes (S1(b), 4.5 mm in thickness) and the bottom part with microstructure pattern (S1(c), 1 mm in thickness). Both parts were made of PC and fabricated by injection molding (RITEK, Taiwan). Two parts were assembled using plasma-enhanced thermal bonding. To create an environment for thermal bonding (pressure and temperature), a thermal bonding system was designed, which including a chamber made by aluminum, an air compressor (Supplementary Figure S2), and an external heat source.

Before assembling the components, there were several treatments for sterilization and removing the dirt on plate spares. First, both components were rinsed by 70% ethanol, then blow dried with filtered air. Afterward, the components were treated with O2 plasma by a plasma generator[1] (CUTE, FEMTO science) for surface modification and plasma-sterilized. After finishing above-mentioned pretreatments, thermal bonding was then applied to assemble two components. First, the components were placed in the chamber following the chamber was heated by a hotplate, 3kgw/cm^2 pressure was pre-applied until temperature reaching 70℃. Afterward, the bonding pressure was increased to 4 kgw/cm^2, maintain the pressure and temperature for 25mins. After that, turned off the hotplate, vented and decreased the pressure to 3 kgw/cm^2. Finally, while the chamber cooled down to room temperature, the chamber was vented to atmospheric pressure and took out the completed MGP from the bonding chamber.

Before usage, the micro-gap plate is UV-sterilized overnight. Then collagen[2](100μg/mL) (C7661, Sigma-Aldrich) was introduced into the culture well to coat on the surface at 37℃ for 1 hour[3,4].

Dilution procedures for uniform cell seeding in the microfluidic device

A uniform cell seeding should be obtained in this assay since the total amount of cells from each tumour is extremely few. In addition, any shift on the quantity of cells among MGP units could considerably affect the outcome of tumour response assay since cell-cell contact would produce more extra-cellular matrix for higher drug resistance[5,6]. To minimize the variation on cell number between MGP units, a two-step dilution operation was applied in the preparation of cell suspension to MGP. The complete operation of preparation was 1. Measure the density of original cell suspension. 2. Determine the times should the cell suspension be diluted (Ex. N times). 3. Dilute the cell suspension into $\sqrt{N}$ times and then the expected density would be obtained after two repeats. 4. After the dilution, density was measured again to determine the corresponding volume of cell suspension to be introduced.

Estimation of cell loss

Cell loss was minimized by removing solution through surrounding microchannels instead of directly through culture wells. To confirm cells were successfully conserved during the operation, quantity of cells before and after solution exchange was compared. A fluorescent dye (Hoechst, H33342) was used to stain cells for better identification. After cells were seeded overnight, fluorescent dye was then introduced to identify the initial number of cells. The optimal cycles of solution exchange were conducted as stated in following section. After completed solution exchange, images were captured to quantify number of cells in each MGP unit. Difference in quantity of cells was then compared and efficiency of this operation was shown in rate of cells remained.

Rate of cells remained = (Initial number-Final number)/(Initial number) X 100%

Repeats for sufficient solution exchange

While considering sufficiently replaced the solution in MGP units, bubbles appearance in MGP would harm the patterned cells. In order to prevent bubbles occur, solution was partially removed in every repeat of solution exchange and the complete operation would be conducted by repeats of exchange. Thus, insufficient repeats of solution exchange may result in unexpected shifts on concentration of reagent in drug treatment and fluorescent staining. In order to confirm that reagents were completely replaced for these two steps, a fluorescent dye (FITC, 50μM in PBS) was used to test the concentration of reagents after repeats of exchange. The intensity of fluorescent dye represented concentration of reagents in MGP units. PBS was firstly introduced into MGP units and to be replaced by FITC. Each repeat of exchange was composed by removing 8μL of PBS from microchannels and introducing the same volume of FITC to culture wells. Pictures of fluorescent dye in MGP units were captured after every repeat of exchange to identify the concentration of FITC in MGP units. After repeats of exchange, the dye-filled MGP was incubated in 37℃ with PBS-filled reservoirs for 24 hours respectively for confirmation of long-term stability of concentration. Fluorescent images were captured by MetaMorph to compare the concentration before and after incubation. Captured images were analyzed by imageJ software for intensity. After the operation stated above, the least repeats of solution exchange was obtained. Supplementary Figure S3 showed the concentration of fluorescent dye (FITC) in the culture well of a MGP unit after every repeat of solution exchange. According to the results, we need to conduct at least 2 repeats for the sufficient solution exchange on MGP, it will take around 30 seconds for one well unit. In addition, concentration of

solution in MGP units was relatively stable during 24 hours of incubation after 2 repeats of solution exchange comparing to 1 repeat of that.

The correlation between manual and auto quantification

The cell quantification in the research was all conducted by a commercialized software, MetaMorph. The software allows cell quantification by setting the minimal and maximal diameter of single stained objects, minimal intensity of objects comparing to local back ground and the minimal and maximal stained area. By applying the auto quantification, quantity of stained cells can be easily and rapidly obtained. In order to apply this tool for quantification of viability assessment in this research, the stained condition should be optimized and strictly followed. On the other hand, while finding the optimal staining condition on the MGP, the optimal settings in MetoMorph were also conducted for accurate cell quantification.

Before applying the quantification method, we should examine the accuracy of auto quantification and also build a correlation between manual count and auto count. The images of fluorescent stained cells were randomly chosen by random number. Three views of each well were chosen to compare the result of manual count and auto count. Manual count was conducted firstly before auto count. Then, auto quantification was conducted by MetaMorph. The results of manual count would not affect the outcome of auto quantification since the number of cells was concluded after processing the quantification in software. Before processing quantification, one could only change the settings in MetaMorph for optimal cell identification. Once the optimal cell identification was found, the auto quantification would then be processed. So, the two methods of quantification would not affect to each other.

Supplementary Figure S4 showed the result of correlation between auto quantification and manual quantification. The number of counted cells was normalized against that of manual count which could directly find the difference between the two methods. Result showed that the weighting factor of auto quantification was 1.0322, which further indicated that the method was averagely 3.2% more than manual

quantification. In addition, no statistical significance between the two methods was found which proved that auto quantification could provide reliable results of quantification in this research.

Morphology of MCF7 and MDA-MB-231 cells during treatment

The morphology of MCF7 cells during 24 hour cisplatin and docetaxel treatment was shown in Supplementary Figure. S5(a) and S5(b) respectively. The seeded cells were incubated overnight prior to drug treatment. After the overnight incubation (t = 0), cells became flat in shape which indicated that cells were well attached to the substrate. At t = 12 hr of drug treatment, cells at higher concentration of drug treatment became rounded and cells treated by docetaxel became detached. At t = 24 hr of drug treatment, the morphology of cells showed flat to rounded from low concentration to high concentration, which was with high consistency with cells treated in 96-well plates. Both the cisplatin-treated and docetaxel-treated cells showed good correlation with those treated in 96-well plates in morphology. In addition, a difference between cicplatin-treated cells and docetaxel-treated cells in morphology was found, which may be caused by different pharmacological mechanisms. Furthermore, docetaxel caused more cell-detachment than cisplatin did on MGP and the same phenomenon was also found in drug response assay on 96-well plates. After the 24 hour drug treatment, all nucleus cells and dead cells were stained by a blue dye (Hoechst) and green dye (SYTOX) respectively to understand the cell viability in different concentrations of anticancer drug. Moreover, cell loss was reduced by applying proper operation of solution exchange so the quantification of viability would be more accurate since all cells were almost kept in units of the MGP. Cell loss could cause over estimation of cell viability since dead cells were easy to get detached from substrates. As a result, the fluorescent signals of detached dead cells could no longer be detected after drug treatment. In short, MGP would conserve almost all cells for higher accuracy of viability assessment.

The morphology of MDA-MB-231 cells during 24 hour cisplatin and docetaxel treatment was shown in Supplementary Figure. S6(a) and S6(b) respectively. At t = 0

hr, the cells were well attached to the substrate and the morphology of cells showed spindle shape which is typical to this cell line. At t = 12 hr, cells became considerably shrunk in higher concentration drug treatment. At t = 24 hr, the cells were stained with a fluorescent dye mixture which contained Hoechst and SYTOX to identify all cells and dead cells respectively. Substantially, the cells treated with higher concentration anticancer more green spots were detected and the morphology of cells showed more rounded than those untreated cells. Both the morphology of cisplatin-treated and docetaxel-treated cells resembled those treated in 96-well plates. In addition, the difference of morphology between concentrations using MDA-MB-231 showed more considerable than that using MCF7 since the morphology of untreated MDA-MB-231 is more slender. Furthermore, a more considerable migration of MDA-MB-231 cells was found comparing to MCF7 cells owing to the innate difference of the two cell types. As a result, the images of treated cells were found slightly different every 12 hour of treatment. On the other hand, comparing with cisplatin-treated MCF7 cells, MDA-MB-231 cells were treated with a further higher concentration (200μg/ml). The reason was that while conducting the same concentration gradient with MCF7 cells on MDA-MB-231 cells, the value of IC_{50} was not available since MDA-MB-231 showed more resistant to cisplatin. As a result, an assay with higher concentration gradient was conducted again to obtain the value of IC_{50}.

Comparisons between the microfluidic culture and conventional cultures

Comparisons with conventional cultures were presented in Supplementary Table S2. Microscale culture in the MGP is slightly different from macro culture in 96-well plate in volume density, surface area to volume ratio. In addition, the shifts on concentration of solution would not be an issue since the volume of working solution was relatively high than microcultures. According to the results conducted on MGP and 96-well control, the response profile of cells treated by different anticancer drugs showed slightly different with each other since the culture environment was different. However, by applying statistical analysis, the results showed no statistical significance

between the platforms which proves that the microculture platform is a viable tool for anticancer drug assay.

1. Mehta, G. et al. Hard Top Soft Bottom Microfluidic Devices for Cell Culture and Chemical Analysis. *Analy. Chem.* **81**, 3714-3722 (2009). doi:Doi 10.1021/Ac802178u.

2. Duffy, D. C., McDonald, J. C., Schueller, O. J. A. & Whitesides, G. M. Rapid prototyping of microfluidic systems in poly(dimethylsiloxane). *Analy. Chem.* **70**, 4974-4984 (1998).

3. Madri, J. A. & Williams, S. K. Capillary Endothelial Cell-Cultures - Phenotypic Modulation by Matrix Components. *J. Cell Biol.* **97**, 153-165 (1983).

4. Folkman, J., Watson, K., Ingber, D. & Hanahan, D. Induction of Angiogenesis during the Transition from Hyperplasia to Neoplasia. *Nature* **339**, 58-61 (1989).

5. St Croix, B. & Kerbel, R. S. Cell adhesion and drug resistance in cancer. *Curr. Opin. Oncol.* **9**, 549-556 (1997).

6. Desoize, B. & Jardillier, J. C. Multicellular resistance: a paradigm for clinical resistance? *Crit. Rev. Oncol. Hemat.* **36**, 193-207 (2000).

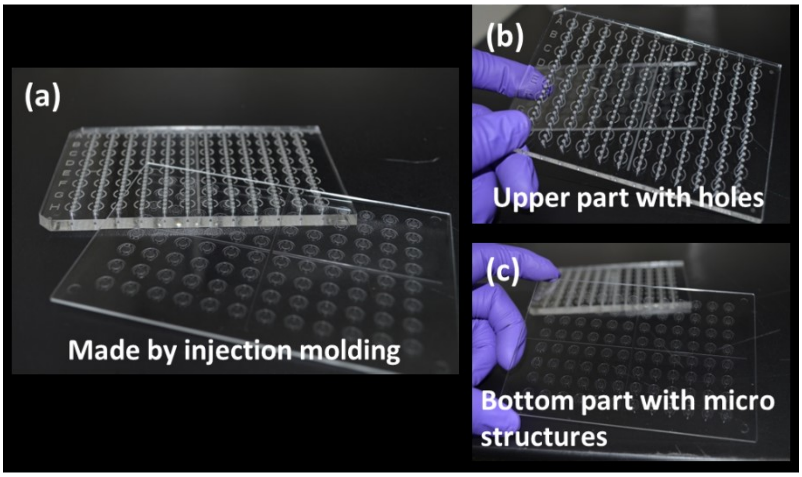

Supplementary Figure S1. The components of the Micro-gap plate (MGP). (a) top part and the bottom part of MGP, (b) top part of MGP with 4.5mm in thickness, (c) bottom part of MGP with microstructures and 1mm in thickness.

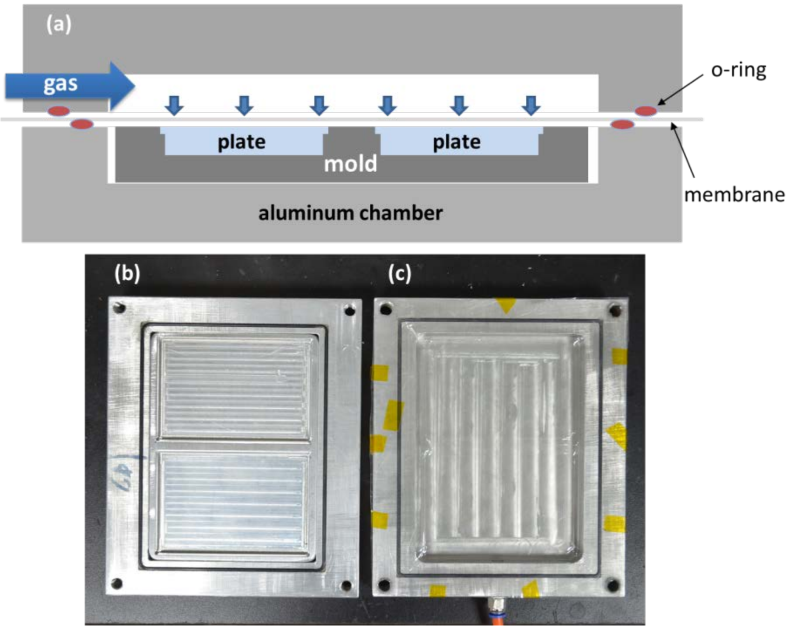

Supplementary Figure S2. Thermal bonding chamber. (a) Cartoon drawing of the bonding chamber applied with pressured air, (b) the bottom part of the chamber, (c) the top part of the chamber.

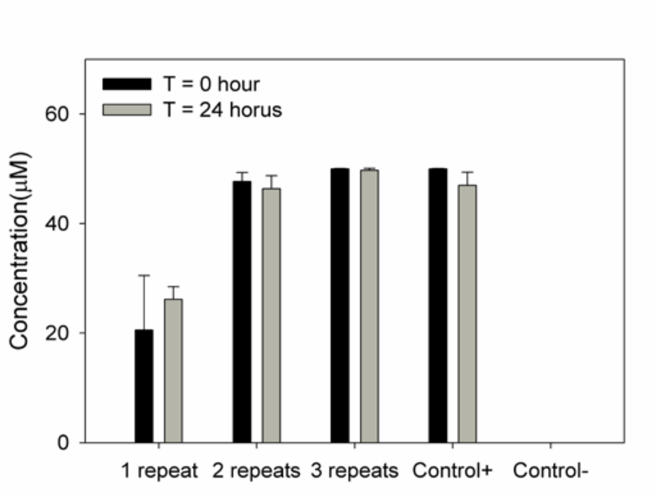

Supplementary Figure S3. Concentration of solution after different repeats of solution exchange. The concentration of FITC was 50μM. As the concentration of FITC obtained to 50μM then the exchange was complete. After at least 2 repeats of solution exchange, the concentration of FITC reached to 50μM. In addition, after 24 hours of incubation, the concentration did not shift statistical significantly. Control+ : The units with 50μM of FITC. Control- :The units with PBS (0μM of RITC) (Data were mean ± standard deviation (SD), n = 3, Student's t-test, $P < 0.05$).

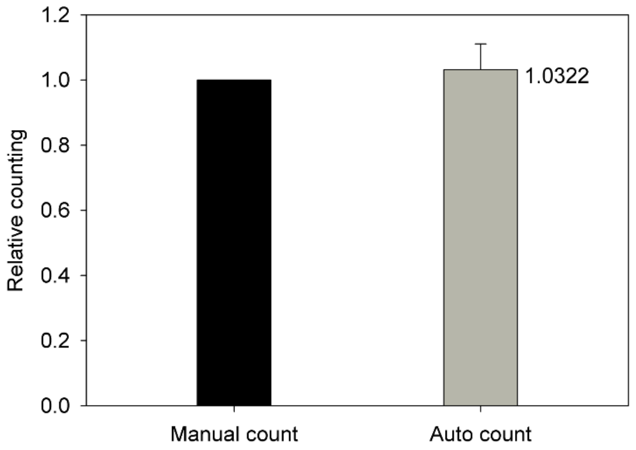

Supplementary Figure S4. The correlation between manual quantification and auto quantification. No statistical significance was found between the two quantification methods. (Data was means ± SD, n = 12, Student's t-test, P > 0.05)

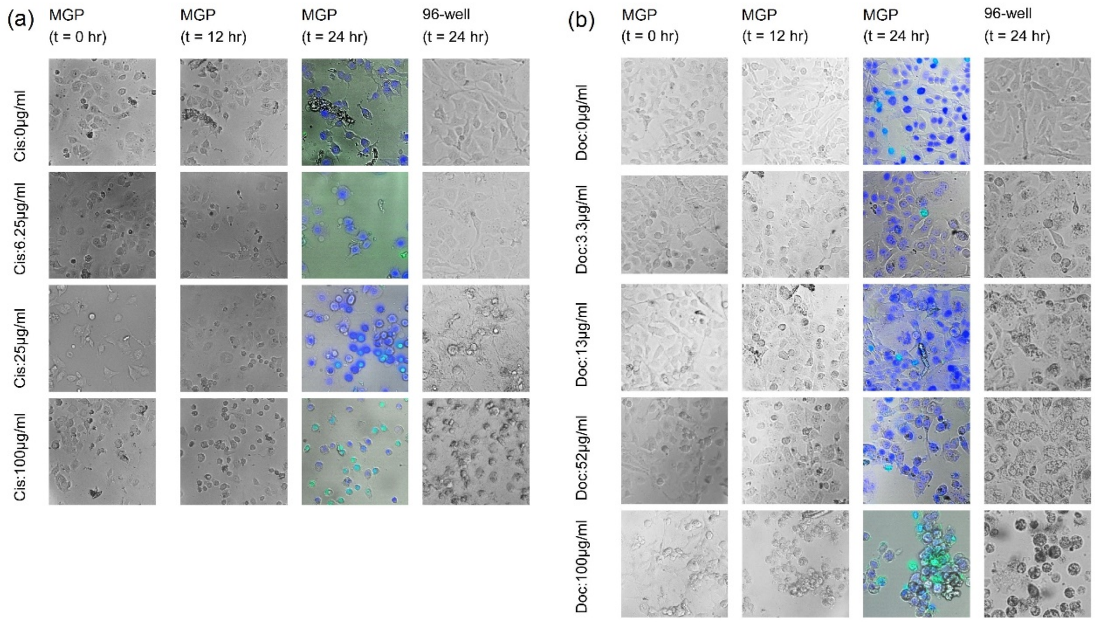

Supplementary Figure S5. Morphology of MCF7 cells during (a)cisplatin and (b)docetaxel treatment. After 24 hour drug treatment, all cells and dead cells were identified by a blue fluorescent dye (Hoechst) and a green fluorescent dye (SYTOX) respectively. Images of cells in 96-well plates were captured as references. In fluorescent images, the cells stained both by Hoechst and SYTOX (dead cells) could be indicated from light blue signals. The number of green spots increased as the concentration of anticancer drugs increased, and the intensity of green dye also rose.

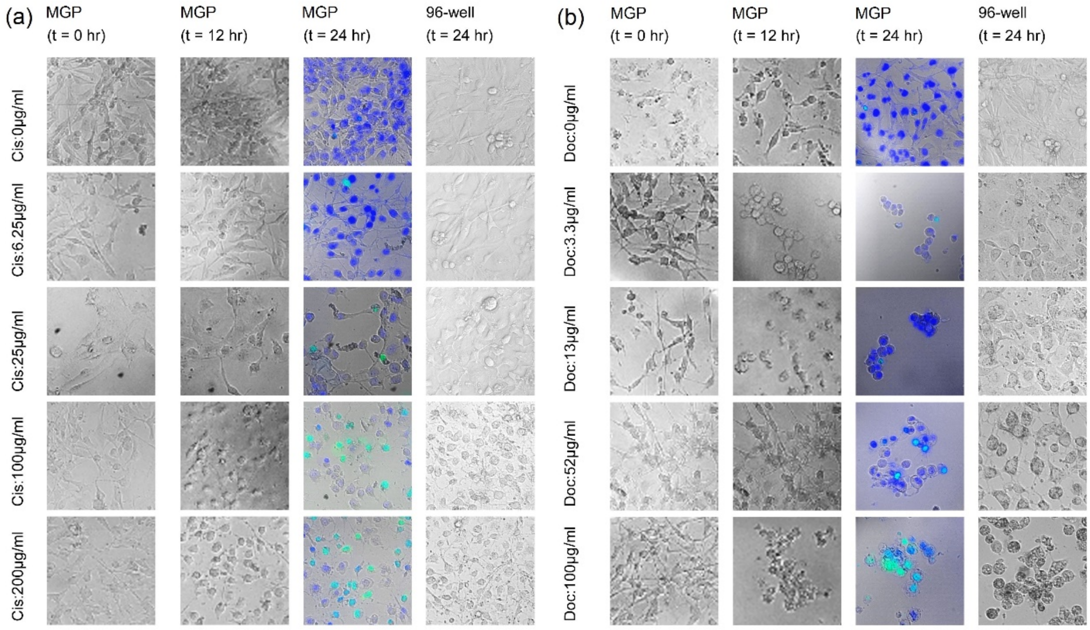

Supplementary Figure S6. Morphology of MDA-MB-231 cells during 24 hour (a)cisplatin and (b)docetaxel treatment. The blue signals stand for all cells while green signals stand for dead cells. The morphology of cells by MGP is similar to those in 96-well plates. Considerable cell detachment was observed in docetaxel test on both plateforms.

Supplementary Table S1. The comparison with existing drug assays

Assay	Method	Cancer types	Assessment Period	Required cell amount	Cancer cell identification	Neoadjuvant prediction	Price
Human tumour cloning system (HTCA)	Treat single concentration of anticancer drug on tumour colony-forming units (TFUs) to find the most effective single treatment.	N/A	14 days of culture to form colonies. Results returned in 3-4 weeks.	$5X10^5$/ml At least 20 colonies(50 cells in per colony)/500,000 cells plated	N	N/A	N/A
Capillary cloning system (CCS)	Form cell colony in agar in tubes. The treatment is same as above.	N/A	Patients waited 10-14 days for results.	$8X10^5$/ml	N	N/A	N/A
Extreme drug resistance assay (EDR)<br><br>＊Oncotech	Find the least effective anticancer drug of individuals. Tritiated thymidine is introduced during the last two days of culture as a measure of cell proliferation.	N/A	Cells incubated with drug for 5 days.	30,000 cells/well	N/A	N/A	N/A

＊: No longer existed

Assay	Method	Cancer types	Assessment Period	Required cell amount	Cancer cell identification	Neoadjuvant prediction	Price
Differential staining cytotoxicity (DiSC)<br><br>Weisenthal cancer group http://weisenthalcancer.com/<br><br>Rational Therapeutics http://www.rational-t.com/	The entire contents of the cell culture are cytocentrifuged onto permanent microscope slides and differentially stained to allow discrimination of normal and neoplastic cells and living and dead cells. Nicrosin stain and H&E stain were applied to viability assessment.	Bladder and Kidney<br>Breast<br>Colorectal<br>Gynecologic<br>Leukemia<br>Lung<br>Lymphoma/NHL<br>Melanoma<br>Pancreatic and Liver	7-10 days	0.5-1g (biopsy)<br>1-2 g (tumour)	Y	Weisenthal cancer group: Do not allow "fine needle aspiration"<br><br>Rational Therapeutics: A needle biopsy is not enough.	$3500~ >5000 Depends on the number of drug used
Subrenal capsule assay (SRCA)	Insert tumour pieces into mouse and treat them with different concentrations of anticancer drugs for days and check the size of tumour in mouse.		Measure the difference of size of tumours between Day0 and Day6.	1mm^3 pieces	No need,	N/A	N/A

Assay	Method	Cancer types	Assessment Period	Required cell amount	Cancer cell identification	Neoadjuvant prediction	Price
Hsitoculture drug response assay (HDRA)<br><br>AntiCancer<br>http://www.anticancer.com/	3-dimensional culture of primary cancer cells in collagen sponge gels.<br>MTT was applied to assess the cell viability.	all Solid Tumour Types	Cells incubated with drug for 7 days and the MTT was introduced for viability assessment.	10-mg tissue on each gel in 24-well plate.	N	N/A	N/A
Microculture Kinetic Assay (MiCK assay)<br><br>DiaTech<br>http://diatech-oncology.com	Microculture Kinetic assay for apoptosis (MiCK assay)<br>The morphology of apoptotic cells can be recognized by time-lapse monitoring the cell morphological change.	Breast<br>Lung<br>Ovarian<br>Sarcoma<br>Leukemia	3-4 days	As more as possible<br>at least 1.5 cm^3	N/A	N/A	$5000

Assay	Method	Cancer types	Assessment Period	Required cell amount	Cancer cell identification	Neoadjuvant prediction	Price
ChemoFx<br><br>PrecisionTherapeutics<br>http://www.chemofx.com	Chemosensitivity was obtained after drug treatment by quantification using fluorescent images (DAPI, only stain the attached cells since the dead cells were hypothesized to be detached).<br>Dose response curve can be obtained.	Bladder<br>Brain<br>Breast<br>Cervical<br>Colorectal<br>Endometrial<br>Gastric<br>Genitourinary<br>Head and Neck<br>Hepatobiliary<br>Kidney<br>Lung<br>Melanoma<br>Ovarian Cancer<br>Pancreatic<br>Penile<br>Prostate<br>Sarcoma<br>Testicular<br>Urinary Tract	3 weeks	At least 35mg of tissue = 2 core biopsies	Y<br>The epithelial cells were identified by staining cocktail of antibodies (AE1/AE3, Cam5.2, CK7 and CK20 was used to identify colon epithelial cells from other.).	**Biopsy specimens:**<br>14g needle; ship a minimum of 2 cores<br>16g needle; ship a minimum of 3 cores<br>18g needle; ship a minimum of 4 cores<br>For vacuum-assisted biopsies, submit 1-2 biopsies	$450 per drug<br>Average $3300

Assay	Method	Cancer types	Assessment Period	Required cell amount	Cancer cell identification	Neoadjuvant prediction	Price
†NexCourse<br>(Resistance enzyme assay)<br><br>GENOPTIX Medical Laboratory<br>http://www.genoptix.com/	Gene expression analysis to find out the possible favorable treatment.	Colon<br>Non-small cell lung cancer	N/A	N/A	No need	N/A	N/A
†OncotypeDX<br><br>Genomic Health.<br>http://www.oncotypedx.com	Examine the expression of 21 genes which are related to tumour growth. Predict the rate of recurrence and the benefit could get from adjuvant chemotherapy.	Breast (StageI and II invasive breast cancer, ER+)<br>Colon	7-10 days	N/A	No need	Perform after surgery.	$4075
MGP assay<br><br>Microfluidics lab, IAM, NTU<br>http://homepage.ntu.edu.tw/~amw/	A microfluidic device was developed for different concentrations of anticancer drugs treatment on primary cells for 24 hours.	Breast, theoretic-ally for all tumour types	3 days	1,000 cells/well<br>⇨ 9,000 cells/drug	Y<br>(CD326 conjugated with PE was applied to identify epithelial cells.)	Y	Cheaper

†: Resistance enzyme assay, measuring the expression of genes coding for specific drug-resistance-related enzymes, measuring the cellular content of these enzymes.

Supplementary Table S2. Comparisons between macroculture and microculture

**Difference**		**96-well plate (macroculture)**	**MGP (microculture)**
Culture scale	Volume of media	100µl (per well)	16µl (per well)
	Total surface area/volume ratio	9.2×10^{-4} µm^{-1} (per well)	2.96×10^{-3} µm^{-1} (per well)
	Surface density	5.65×10^{-4} cells µm^{-2}	5.68×10^{-4} cells µm^{-2}
	Volume density	170 cells µl^{-1}	62.5 cells µl^{-1}
	Required cell amount	17,000 cells (per well)	1,000 cells (per well)
